# Clinical Signs, Advanced Diagnostic Imaging Findings, Treatment, and Outcome of Mycotic Discospondylitis in 11 Dogs

**DOI:** 10.1111/jvim.70097

**Published:** 2025-04-24

**Authors:** Samuel Okonji, Andrea Di Paola, Silvia Benini, Antonella Gallucci, Alberto Cauduro, Cristian Falzone, Teresa Gagliardo, Gualtiero Gandini

**Affiliations:** ^1^ Dipartimento di Scienze Mediche Veterinarie, Department of Veterinary Medical Sciences Università degli Studi di Bologna Ozzano dell'Emilia Italy; ^2^ Neurovet Milano Italy; ^3^ Centro Veterinario Professionale La Fenice Assemini Italy; ^4^ Clinica Veterinaria Pedrani Diagnostica Piccoli Animali Srl Thiene Italy; ^5^ Diagnostic Center Palermovet Palermo Italy

**Keywords:** antimycotic treatment, aspergillosis, diskospondylitis, fungal, microbiology, systemic mycosis

## Abstract

**Background:**

Discospondylitis refers to inflammation of the intervertebral disc and adjacent vertebral endplates. The literature on mycotic discospondylitis (MD) in dogs is limited.

**Objective:**

To describe clinical and advanced diagnostic imaging findings, therapeutic strategies, and outcomes in dogs with a confirmed diagnosis of MD.

**Animals:**

Eleven client‐owned dogs with a diagnosis of MD.

**Materials and Methods:**

Medical records from five veterinary neurological referral centers were retrospectively reviewed between 2017 and 2024. The confirmed diagnosis of MD was based on clinical and magnetic resonance imaging (MRI) findings and the detection of fungal hyphae in urine, intervertebral disc, or cerebrospinal fluid (CSF).

**Results:**

German shepherd (GS) were the most prevalent breed (7/11). Pain was the main clinical sign reported in all dogs, associated with gait abnormalities in 9 dogs. T3‐L3 neuroanatomical localization was described in 10 dogs. MRI showed multiple intervertebral disc involvement in 7 dogs. Fungal hyphae were identified in urine sediment in 5 dogs and by CT‐guided needle aspiration of the affected disc in 2 dogs. *Aspergillus* spp. was the most common etiological agent being reported in 7 dogs. Ten dogs were dead at the end of data analysis, with a median survival time of 30 days.

**Conclusion and Clinical Importance:**

This case series demonstrates the necessity of accurate diagnosis to set an appropriate treatment, despite the poor prognosis after antifungal therapy.

**Abbreviations**
CNS: central nervous system
CRP: C‐reactive protein
CSF: cerebrospinal fluid
CT: computed tomography
GSs: German shepherds
MD: mycotic discospondylitis
MRI: magnetic resonance image
T1W: T1‐weighted
T2W: T2‐weighted

## 1 Introduction

Discospondylitis is an infection of the intervertebral disc and the adjacent vertebral endplates [1]. It is well recognized in human medicine and is described in the veterinary literature in domestic animals, including dogs, cats, horses, cattle, and swine [2, 3, 4]. The disease is considered common in dogs. Publications on discospondylitis include three studies of 513, 386, and 120 dogs [5, 6, 7]. Discospondylitis is more frequently diagnosed in medium‐to giant‐breed dogs of all ages, with the most common localization of infection being at the level of the lumbo‐sacral junction [8]. The most common route of infection is hematogenous spread from a primary site such as the genitourinary system, skin, and mouth. Less commonly, direct infection secondary to a migrating foreign body or previous surgery is reported [9].

Bacterial etiology is by far the most common cause of discospondylitis in dogs and is generally associated with a good prognosis for recovery [1, 9], with a range from 76% to 86% of dogs achieving complete resolution of clinical signs [6, 7, 10]. Rare reports of mycotic infections are described in veterinary medicine, all associated with a poor outcome [11, 12, 13, 14].

German shepherds (GSs) appeared to be predisposed to mycotic discospondylitis (MD) in one study [11]. *Aspergillus* spp., a saprophytic and ubiquitous organism, is the most commonly diagnosed fungus [8]. Other fungi reported as causative agents of discospondylitis in dogs include *Pseudallescheria boydii*, *Scedosporium apiospermum*, *Coccidioides immitis*, *Mucor*, *Fusarium*, *Paecilomyces*, *Penicillium*, and *Chrysosporium* spp. [9, 12, 14] Reported treatment consists of administration of antifungal drugs such as itraconazole and fluconazole, and despite prolonged treatment, sometimes for life, the prognosis is poor [9, 10, 11, 12]. *Aspergillus* spp. is resistant to fluconazole.

Currently, there is limited literature on MD. Except for rare case reports [9, 10, 11, 12, 15], the study with the largest cohort (10 dogs) was published approximately 30 years ago and included limited information on signs of neurological disease. It should be considered that diagnostic imaging in this case series consisted only of plain radiography due to technological limitations. The aim of this study is to contribute to the knowledge of MD in dogs by describing the clinical and advanced diagnostic imaging findings, therapeutic approach, and outcome in a cohort of 11 dogs.

## 2 Materials and Methods

Cases were identified retrospectively by searching all parts of the medical records of five veterinary referral centers using the following terms: “MD”, “fungal discospondylitis,” and “systemic mycosis,” from 2017 to 2024.

Dogs were diagnosed by a board‐certified veterinary neurologist (ECVN diplomate) or ECVN resident.

In order to be included in the study, dogs were required to meet all of the following inclusion criteria:

- Information on the neurological condition, including neuroanatomical localization of the lesion.
- Findings of advanced imaging diagnostic techniques (Computed tomography [CT] or magnetic resonance imaging [MRI]) compatible with discospondylitis. Specifically, MRI findings include morphological and signal changes of the intervertebral disc, endplates, and vertebral body (hyperintense signal on T2‐weighted [T2W] sequences, hyperintense on STIR sequences, iso‐hypointense on T1‐weighted [T1W] sequences, and enhancement after intravenous administration of paramagnetic contrast) and CT findings include osteolytic changes at the level of the endplates and vertebral bodies with reduction of the affected intervertebral space, subchondral sclerosis, and osteoproliferative lesions;
- Detection of fungal hyphae on cytological examination of the urine sediment (collected by cystocentesis), intervertebral disc, or cerebrospinal fluid (CSF).
- Information on the follow‐up is available on the clinical records or telephone call to the owner.

Dogs were excluded if there was incomplete information on neurological examination results and neuroanatomical localization, if they had not undergone advanced diagnostic imaging, or if there was no information on the outcome.

Data collected from the dogs records included age at presentation, sex, neuter status, breed, body weight, onset, progression, and duration of clinical signs before presentation, presence of systemic clinical signs, neurological status, and neuroanatomical localization (C1‐C5, C6‐T2, T3‐L3, L4‐S3 or multifocal), blood test results (hematology and serum biochemistry including C‐reactive protein [CRP]), urinalysis, mycological culture, diagnostic imaging findings (MRI and/or CT and, in selected cases, radiographs of the vertebral column, abdominal ultrasound, and chest radiographs), treatment administered after diagnosis, survival time (from the time of etiological diagnosis to death), and outcome. Data on treatment protocols were recorded, including type of antimycotic drug, dosage, duration of treatment, and any other medication. The presence of elongated extradural material T1W iso‐hypointense with enhancement after administration of intravenous paramagnetic contrast was recorded as a concomitant lesion indicative of spinal epidural empyema.

Cerebrospinal fluid (CSF) examination with assessment of protein content and cell count, and cytology was included if performed.

Neurological status was defined according to the Modified Frankel scale (MFS) as follows: 5 (paraplegic, deep pain negative); 4 (paraplegic deep pain positive); 3 (non‐ambulatory paretic); 2 (ambulatory paretic); 1 (spinal pain only); 0 (neurologically normal) [16].

The clinical course of discospondylitis was classified as acute if < 2 days, subacute if between 2 and 7 days, and chronic if > 7 days, based on the time from the onset of signs to the diagnosis of mycotic infection. Progression was assessed by comparing the neurological findings at the initial neurological examination with the follow‐up neurological examination, including assessment of gait, postural reaction, and spinal pain, and categorized as static, worsening, improving, or intermittent. Response to treatment was evaluated using the same timeline and variables as for progression and was classified as positive if there was a clinical improvement (at least by 1 grade), stable if there was no improvement, or negative if there was worsening of clinical signs.

Relapse was defined as a recurrence of clinical signs after the improvement or the achievement of a normal neurological condition.

The reason for the death of the dogs and whether it was related to the mycosis was recorded.

The collected data were recorded and analyzed in a spreadsheet using Microsoft Excel for Mac version 16.74. Statistical analysis was descriptive, and continuous variables such as age and body weight were described using the median value.

## 3 Results

### 3.1 Animals

**FIGURE 1:**
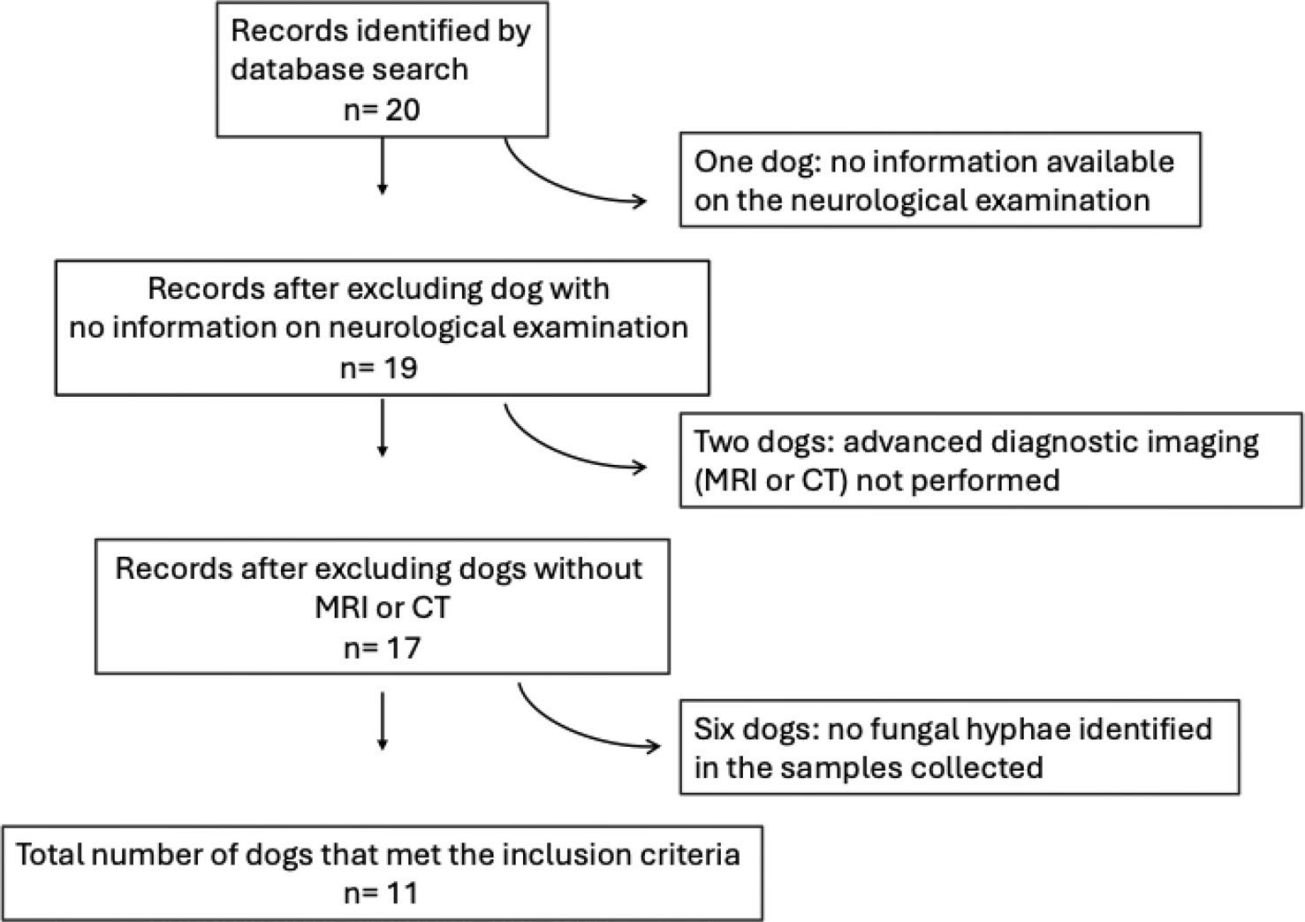
Study protocol for case selection. *n*, Number of dogs.

Sixteen dogs with presumed MD were identified from the database search. One case was excluded due to a lack of information on neurological examination. In two dogs, no advanced imaging was performed, and in two cases, no fungal hyphae were identified in the samples collected. A total of 11 dogs met the inclusion criteria (Figure 1).

Details data on signalment, onset, and progression of clinical signs, neurological status, and neuroanatomical localization of the study cohort are provided in Table S1 of the Supporting Information.

The median age of the enrolled dogs was 5.1 years (range 3.2–10.9 years), and the median weight was 32.5 kg (range 16–48 kg). Six dogs were females (4 entire and 2 neutered), and 5 dogs were males (4 entire and 1 neutered).

The most represented breed was the GS, with a total of seven dogs. The other breeds represented were Siberian Husky (one dog), Belgian Shepherd (one dog), Bull terrier (one dog), and mixed breed dog (one dog).

### 3.2 Anamnesis

The main complaint of the owners was ambulatory deficits, reported in nine dogs and associated with overt pain in 2. In the other two cases, owners reported only pain without obvious gait abnormalities.

Regarding the presence of previous illness, two dogs had a urinary tract infection (of which one treated with enrofloxacin for 1 week) and 1 dog had undergone ovariohysterectomy 2 weeks before due to a pyometra. Further and more specific information was not provided by the referring veterinarians.

The course of discospondylitis was chronic in 9 (81.8%) dogs, acute in 1, and subacute in another (9.1%) case. Nine dogs had progressive signs, while in 2 dogs, signs were intermittent.

### 3.3 Clinical Signs, Neuroanatomical Localization, and Blood Test Results

Information on general physical examination findings was available for 10 dogs. Out of these, 4 dogs showed no abnormalities. Six (54.5%) dogs showed pyrexia with a mean temperature of 39.8°C (range 39.5°C–40.1°C) and included 1 dog who also presented with progressive weight loss and anorexia.

The most common clinical finding on neurological examination was pain on palpation of the vertebral column, present in all dogs.

Gait abnormalities were found in 9 dogs. Of these, three dogs had proprioceptive ataxia of the hind limbs and ambulatory paraparesis (grade 2), three dogs had non‐ambulatory paraparesis (grade 3), two dogs were paraplegic with intact nociception (grade 4), and one case was paraplegic with absent nociception (grade 5).

Overall, postural reactions were absent in 6 dogs, with three dogs scoring grade 3 on the MFS, two dogs scoring grade 4, and one dog scoring grade 5.

Postural reactions were reduced in 3 dogs (scoring grade 2 on MFS) and normal in 2 dogs that had no gait abnormalities but only pain on palpation of the vertebral column.

A T3‐L3 neuroanatomical localization was detected in most of the cases (10 dogs). In 1 dog, neurological clinical signs consistent with multiple localizations were found. They included ambulatory changes due to a T3‐L3 spinal cord lesion and pain at the level of the lumbo‐sacral junction.

Blood test results were available for all dogs. The complete blood cell count showed neutrophilic leukocytosis in six dogs, two of which occurred with monocytosis. Serum biochemistry was normal in 5 dogs. Six dogs showed higher CRP, occurring in all cases with neutrophilic leukocytosis. In one dog, abnormal CRP was associated with an elevation in total protein and globulins, and in one case with an abnormally high level of creatinine. In one dog, there was only an abnormally high level of alkaline phosphatase (ALP) activity and blood urea concentration.

### 3.4 Diagnostic Imaging

Two imaging modalities were performed in 7 cases (six dogs underwent radiographs of the vertebral column prior to advanced diagnostic imaging, and one dog underwent a CT of the entire vertebral column after the MRI) while 1 dog underwent three diagnostic imaging modalities (radiographs prior to MRI, and CT of the vertebral column). Three dogs had only MRI.

MRI was performed in all dogs, and information on the localization of the affected area was recorded in all cases. MRI showed lesions involving only one disc in 4 cases, all at the T3‐L3 level, and more than one disc in 7 dogs, four of which involved only the thoracolumbar vertebral column and three with localization at both the T3‐L3 and L4‐S3 levels (Table S2). In the whole population, the median number of affected intervertebral disc spaces was 2.5 (range 1–10). When considering only dogs with multiple sites of discospondylitis, the median number of discs affected was 4.5 (range 2–10).

Lesions compatible with spinal epidural empyema were described in 5 cases, four at the level of T3‐L3 and one at L4‐S3 spinal cord segments. Paravertebral muscle involvement, likely secondary to the infection, was detected in all dogs. MRI findings of a MD associated with an empyema are shown in Figure S1 in the Supporting Information.

Of the 2 dogs that underwent CT, multiple lesions were described in one case. Neither case had lesions compatible with spinal epidural empyema. In these two dogs, the MRI and CT findings were consistent, including the absence of spinal epidural empyema.

Radiographic findings were recorded in 7 dogs. Five dogs had features compatible with discospondylitis, including four with focal lesions and one with multiple discs affected. Abnormalities were not detected on radiographs in 2 dogs. Of these seven dogs with radiographs, all underwent MRI, and one also underwent CT. Two dogs with no radiographic abnormalities had MRI findings compatible with discospondylitis (one with multiple discs involved, and the other dog with a single site of discospondylitis), whereas two of the four dogs with radiographic findings consistent with a single lesion had multiple discs affected on MRI. Comparison between the localization of discospondylitis between MRI, CT, and radiographs is provided in Table S2 in the Supporting Information.

One dog had a CSF sample collected. In this dog, fungal hyphae were present. Information regarding total cell count and total protein was not available.

### 3.5 Diagnosis of Mycotic Infection

The diagnosis of mycotic infection was made in five dogs by the detection of fungal hyphae in the urine sediment. Figure S2 in the Supporting Information shows the presence of fungal hyphae in a urine sample. In 2 cases, the diagnosis was made by cytological examination of material obtained by CT‐guided needle aspiration of the affected disc. In 3 dogs, the diagnosis was made by biopsy obtained by surgical curettage of the infected disc, while in 1 case, fungal hyphae were found on cytological examination of the CSF.

Mycological culture was performed in all dogs from the same samples in which fungal hyphae were found cytologically and the etiological agent was identified in 10 dogs. Specifically, 7 belonged to *Aspergillus* spp. (of which four detected from urine, two by surgical curettage and one by CT‐guided needle aspiration), 1 to *Candida albicans* (identified from urine), 1 to *Saccaromyces* spp. (detected by CT‐guided needle aspiration), and 1 to *Penicillium* spp. (identified by surgical curettage). In one case (the one in which fungal hyphae were found on cytological examination of the CSF), the fungus was confirmed but not identified due to a laboratory issue. Of those belonging to *Aspergillus* spp., it was possible to identify the species (*Aspergillus fumigatus*) in two cases. Detailed information is provided in Table S3 in the Supporting Information.

### 3.6 Treatment

Information regarding treatment was available for 10 dogs. Of these, one dog was not treated because of the onset of acute renal failure shortly after diagnosis and the owner's decision to euthanize the dog. Therefore, 9 dogs received specific antimycotic therapy.

The most commonly used antimycotic drug was itraconazole (Itraconazole, EG S.p.a., Milan, Italy) administered to 7 dogs at a dose of 5 mg/kg per os (PO) q12 h. The other antimycotic agents used were fluconazole (EG S.p.a., Milan, Italy) at 5 mg/kg PO q12h in 1 dog, and amphotericin B (Fungizone, AVAS Pharmaceuticals S.r.l., Milan, Italy) in combination with flucytosine (Ancotil, Meda Pharma S.p.a., Milan, Italy) in another dog. In this latter case, the dose used was not detailed in the clinical record.

Six dogs had a good initial temporary response to the therapy. One dog showed a sustained good response over time and was alive at the moment of the data collection. Two dogs had no response.

Additional therapies used in combination with antifungals were analgesics such as gabapentin (Gabapentin, Teva Italia S.r.l., Milano, Italy) and tramadol (Altadol, Formevet S.r.l., Milan, Italy) in 9 (100%) cases, antibiotics (amoxicillin‐clavulanic acid [Synulox, Zoetis Italy S.r.l., Modena] and marbofloxacin [Aristos, Fatro S.p.a., Bolonia]) in 7 (77.8%) dogs, nonsteroidal anti‐inflammatory (NSAIDs) drugs in 4 dogs, and antiepileptic drugs such as phenobarbital (Soliphen, Dechra Veterinary Products, Turin, Italy), levetiracetam (Keppra, GlaxoSmithKline, Jedda, Kingdom of Saudi Arabia or Matever, Pharmathen, Attica, Greece) and midazolam (Midazolam, Hameln Pharmaceuticals, Hamelin, Germany) in 1 dog due to the development of status epilepticus.

Surgical curettage of the affected disc was performed in 3 cases.

### 3.7 Outcome

At the end of data analysis, 10 dogs had died. The median survival time was 30 days (range 5–365). One dog was still alive and receiving antimycotic therapy 1210 days after diagnosis. The blood test changes, etiological agent, antifungal therapy, outcome, and survival time in our study cohort are summarized in Table S3.

The cause of death was euthanasia in 8 dogs, four due to severe worsening of ambulatory deficits, three due to development of systemic signs (diffuse pneumonia in one dog, signs of systemic mycosis in one case, and acute nephropathy in the other dog), and one dog due to refractory status epilepticus occurring soon after the neurological examination and diagnosis of MD. An unrelated cause (hit by car) was recorded in 1 dog, while death related to an unknown cause was described in another dog.

Follow‐up MRI was performed in 2 cases. One dog underwent a first follow‐up MRI 270 days after diagnosis. MRI showed no contrast enhancement of the previously affected intervertebral discs. After an acute worsening of the clinical signs, a second follow‐up MRI was performed 110 days after the first follow‐up MRI, which showed a severe recurrence of the discospondylitis in the same intervertebral spaces. The dog was euthanized 5 days later due to the gait deterioration.

In the only dog still alive, a follow‐up MRI was performed 510 days after the diagnosis of MD to assess the disease progression. The MRI showed substantial improvement of the previously affected intervertebral discs and endplates without contrast enhancement.

## 4 Discussion

The present retrospective study described 11 dogs with MD, primarily affecting GSs, and found that spinal pain and gait abnormalities were the most commonly reported clinical signs (100% and 82% of dogs, respectively). Despite treatment, the median survival was 30 days. At the time of writing, only one dog was still alive, highlighting the poor prognosis of the disease.

This case series systematically addresses the clinical, advanced imaging, therapeutic, and outcome aspects of dogs with MD. The largest case series on the topic, published when advanced diagnostic imaging was not available in veterinary medicine, lacks detailed information on the diagnosis and treatment [17].

In the English language literature on peer‐reviewed journals, a total number of fewer than 50 dogs with MD as the primary site of infection or because of disseminated disease is described [7, 11, 14, 15, 17, 18, 19, 20, 21, 22, 23, 24, 25, 26]. MRI findings are described in one paper, which included seven cases of central nervous system (CNS) aspergillosis [20].

In agreement with what is reported in the literature, our data showed that the majority (7 of 11) of dogs were GSs. A predisposing factor for aspergillosis in GS is reported by some authors and might include a defect in mucosal immunity, which could have a genetic basis [17, 18]. An inherited immune disorder associated with abnormalities in IgA function is reported in GSs with low IgA serum levels, a finding described in this breed [26]. However, not all GSs with systemic fungal disease have lower IgA levels [27]; therefore, a possible multifactorial etiology should be considered, including immunosuppressive conditions such as diabetes mellitus, chemotherapy treatment, glucocorticoid treatment, or concurrent infection [28]. In our study, the presence of pre‐existing disease in two GSs (one dog with a history of urinary tract infection and one that underwent ovariohysterectomy for the presence of pyometra) suggested possible conditions causing immunosuppression.

While previous literature shows an overrepresentation of males for bacterial discospondylitis [5, 29], females are more reported in MD [17, 18]. Despite the small number of dogs in our study, a slight majority of females was found (6 of 11).

On general physical examination, pyrexia was the most frequent finding, described in six cases. A recent retrospective study involving 120 dogs diagnosed with bacterial discospondylitis confirmed pyrexia in 23% of the dogs [7]. In the published reports on MD, although detailed information regarding the measurement of body temperature is not present in all papers, pyrexia was reported in 11 out of 26 dogs [11, 14, 15, 17, 18, 21, 23, 25, 30, 31].

Neurological examination showed pain on palpation of the vertebral column in all dogs. Nine dogs had gait abnormalities associated with abnormal postural reactions in the hind limbs, suggesting direct spinal cord involvement.

In the presence of discospondylitis, spinal cord damage can be the consequence of compression due to an empyema [32] or, according to the human literature, vertebral instability possibly related to subluxation or pathologic fracture [33].

In our study, out of the nine dogs that showed gait deficits and abnormal postural reactions, none had MRI findings of compressive myelopathy secondary to vertebral instability or pathologic fracture. Among these nine dogs, five had MRI findings suggestive of compressive myelitis due to empyema. The four remaining dogs had no signs of myelopathy on MRI.

In addition, the MRI of one of the two dogs that presented only with pain showed a mild compressive myelopathy in the absence of obvious gait abnormalities.

Direct compression of neural tissue in the vertebral canal and formation of ischemic injuries secondary to thrombosis or vasculitis have been proposed to explain the pathophysiology underlying the sign of neurological disease observed in dogs with spinal epidural empyema [32, 33].

The reason for the gait abnormalities and altered postural reactions in the absence of relevant spinal cord lesions on MRI is not yet completely understood. In a study examining spinal cord compression associated with bacterial discospondylitis, the degree of spinal cord compression did not correlate with the severity of neurologic signs [29].

Twenty‐four hours after the neurological examination, one dog presented with forebrain clinical signs with the onset of a refractory status epilepticus. Although brain MRI and necropsy were not performed, the primary suspicion was for mycotic extension of MD to the intracranial nervous system. Although intracranial spread of nasal aspergillosis through the cribriform plate of the ethmoid is the most likely form of diffusion of this microorganism into the intracranial CNS [24], there are reports of hematogenous diffusion from primary sites of discospondylitis [20], as suspected in this dog. Considering the direction of CSF flow and the lack of progression of signs in the cranial part of the spinal cord, diffusion from the spinal cord to the intracranial structures via CSF seems very unlikely. Although this is a rare condition, a careful evaluation of the mental status and cranial nerves during the neurological examination in dogs with MD is warranted to provide information on possible intracranial involvement, to be confirmed with brain MRI and CSF examination.

Signs of systemic inflammation in blood exams, such as neutrophilic leukocytosis associated with CRP increase, were found in 50% of our cohort. A retrospective study on 16 dogs diagnosed with bacterial discospondylitis showed that CRP assessment might be clinically more useful to screen this disease than pyrexia or leukocytosis alone [34]. As the findings of physical and neurological examination can often be non‐specific, CRP evaluation can be considered in the diagnostic work‐up of dogs with suspected MD. Further studies, including a larger number of dogs, are needed to evaluate whether CRP may be a useful biomarker in the diagnosis of canine MD. MRI is the gold standard in the diagnosis of discospondylitis because it provides more detailed information and can detect lesions earlier than radiographs [35]. In our study, the comparison of the number of sites affected by MD between the different imaging modalities showed that MRI, as in the case of bacterial discospondylitis, is more precise in detecting intervertebral disc infections at an earlier stage even in the presence of mycotic etiologies [36]. In fact, two cases with normal radiographs had lesions compatible with discospondylitis on MRI, and two out of four dogs with radiographic findings consistent with single lesions had multiple discs involved on MRI.

On diagnostic imaging, lesions in affected dogs were predominantly multifocal with a median number of affected discs of 2.5. All dogs had discospondylitis affecting at least the thoracolumbar vertebral column. Although these data seem to confirm what has been reported by other authors [11, 17], it is difficult to compare our findings with the existing literature. In particular, most of the previous papers, especially the less recent ones, describe only the radiographic findings with a possible underestimation of the number and location of affected intervertebral discs. However, our results are consistent with what is reported in the current veterinary literature that MD is more often multifocal than bacterial discospondylitis, for which multifocal localization is reported in 20%–37% of cases based on different studies [6, 7, 11, 15, 17, 18]. Of note, three dogs in our case series had a single lesion.

Additional imaging features to help differentiate between bacterial and MD are not currently available in veterinary medicine. In human medicine, the presence of a focal paravertebral soft tissue abnormality on MRI is most commonly associated with MD [37]. In our study cohort, we found the presence of paravertebral soft tissue abnormalities in 100% of dogs. In a previous study, reporting MRI features of 13 dogs with confirmed bacterial discospondylitis, paravertebral muscle involvement was described in all cases [36]. These results do not appear to support the hypothesis that paravertebral soft tissue involvement might help differentiate mycotic from bacterial discospondylitis as in humans.

In our case series, the diagnosis of MD was obtained by cytological identification of the fungal hyphae and, in all but one case, by mycological culture. The use of enzyme immunoassay tests for the diagnosis of systemic aspergillosis is reported in literature [38]. Although it was not used in our case series and there are no reports of its use in the previous MD literature, it may be of interest to evaluate its usefulness both in refining the diagnosis and in assessing the response to therapy.

The most commonly used antifungal drug in this study was itraconazole, administered in seven dogs. Itraconazole is a synthetic broad‐spectrum azole derivative with more effective in vitro activity than ketoconazole against Candida species, *Aspergillus* species, and dermatophytes [28]. Specific guidelines for antifungal therapy in dogs with MD are not available, but given the intrinsic resistance of *Aspergillus* spp. to fluconazole, it should not be recommended to treat MD in the absence of clear indications from the antimycogram [28].

In human beings, a randomized clinical trial in humans with aspergillosis showed that voriconazole improves survival and is well tolerated compared to amphotericin B [39]. In our country, the prohibitive cost of voriconazole for long‐term administration and the difficulty in obtaining this drug were the reasons for not using this antifungal in our case series.

The results of our study are consistent with the reported poor prognosis associated with this disease [11, 18] despite early diagnosis and treatment. At the end of the data analysis, only one dog was alive 1210 days after the diagnosis of MD and the overall median survival time was 30 days. It is difficult to make a comparison with previous literature as many studies include MD in a broader cohort of dogs affected by systemic mycoses.

The present study has a few minor limitations, mainly due to its retrospective nature and the rarity of the disease. Limitations include the small number of dogs and, considering the retrospective analysis, the lack of a precise timeline for progression and response to treatment. The lack of a standardized therapeutic approach may have influenced individual survival times and relapse rates.

Another limitation might be related to the fact that MRI was performed based on the clinical neuroanatomical localization and, once lesions were found, the entire vertebral column was not further examined, which might have led to an underestimation of the total number of affected sites.

Among the limitations, six dogs were diagnosed with MD based on the detection of fungal hyphae in the urine. The presence of fungal hyphae on urinalysis, together with the presence of lesions on MRI that are not specific for fungal infection, does not prove that the cause of discospondylitis is mycotic, but it makes this hypothesis the most likely. Finally, antimycogram was not performed in our study. Antimycogram might play a role in the selection of the most appropriate antifungal drug and possibly change the survival time.

In conclusion, the results of our study showed that the diagnosis of MD can be challenging because clinical signs and advanced imaging findings are non‐specific, especially in regard to bacterial discospondylitis, and require confirmation by fungal identification. In our study cohort, middle‐aged GS were overrepresented, and it would be important to consider a fungal agent among the causative agents of discospondylitis in this breed. The MD characteristic clinical sign was pain in the affected part of the vertebral column detected in all dogs, occurring with ambulatory deficits of insidious onset in nine dogs. In our case series, the most common etiological agent responsible for the disease, reported in seven dogs, was *Aspergillus* spp. The prognosis was poor despite antifungal therapy.

The results of this study also emphasize that advanced diagnostic imaging does not provide specific information to differentiate between bacterial and MD. The finding of fungal hyphae in the urine sediment should reinforce the suspicion of MD, confirming the need for further diagnostic tests such as a fine needle aspiration or surgical curettage of the affected disc to reach an etiological diagnosis.

## Disclosure

Authors declare no off‐label use of antimicrobials.

## Ethics Statement

Authors declare no Institutional Animal Care and Use Committee or other approval was needed. Authors declare human ethics approval was not needed.

## Conflicts of Interest

The authors declare no conflicts of interest.

## Supporting information


Figures S1–S2.

Tables S1–S3.
