# Supplementary material for: Clinical Signs, Advanced Diagnostic Imaging Findings, Treatment, and Outcome of Mycotic Discospondylitis in 11 Dogs
Source: J Vet Intern Med. 2025 Apr 24;39(3):e70097. doi: 10.1111/jvim.70097 (PMC12018769; doi:10.1111/jvim.70097)
Supplement: Supplementary file 1 — Figures S1–S2. [file JVIM-39-e70097-s002.pdf]

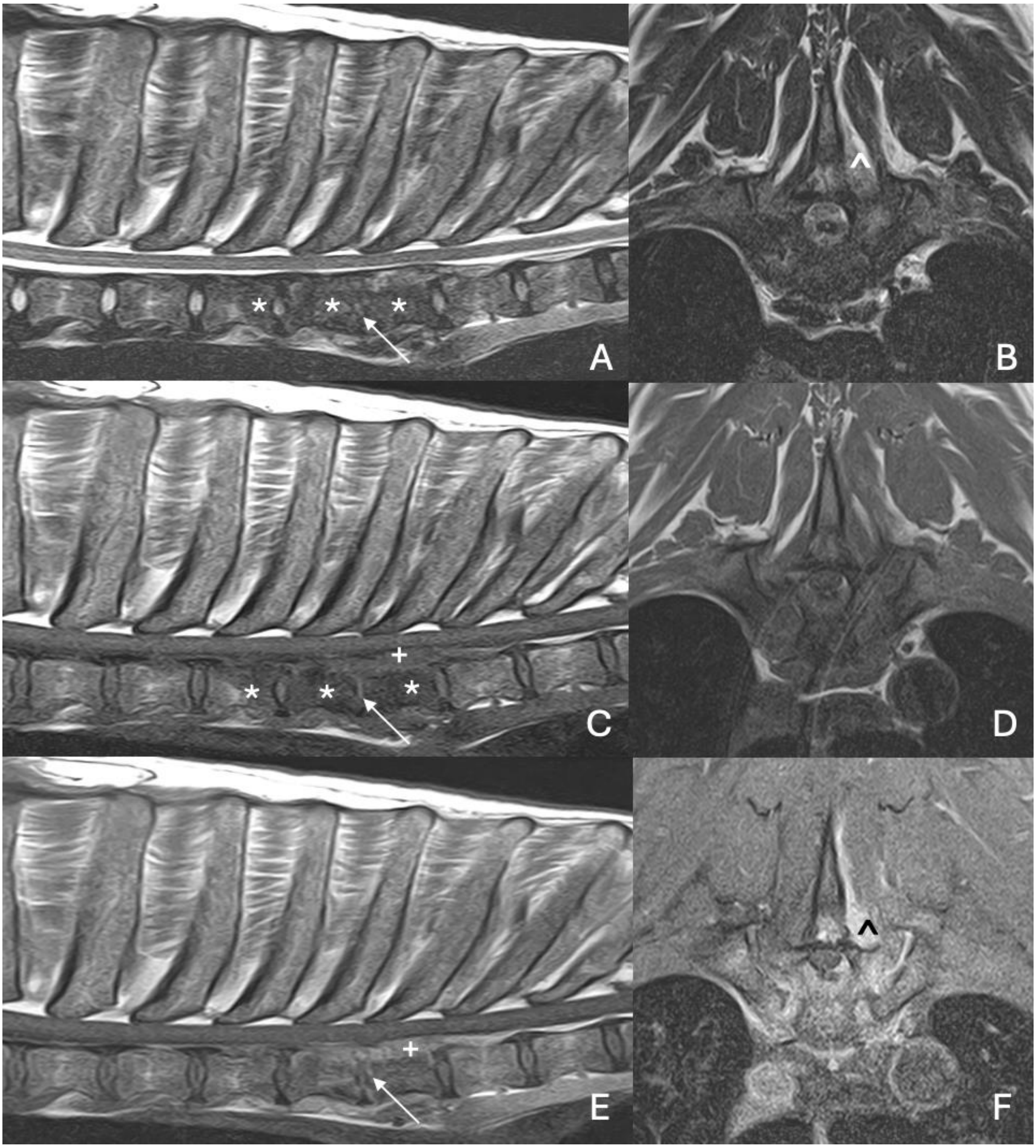

FIGURE 1

MRI features of dog 1 showing multiple sites of discospondylitis with an epidural empyema. Sagittal (A) and transverse (B) T2W images; sagittal (C) and transverse (D) T1W images; sagittal (E) T1W post-contrast and transverse (F) T1W FAT SAT post-contrast images. A, C and E: sagittal images of the T2-T8 vertebral column; B, D and F transverse images are centered at the level of the T5-T6 intervertebral disc.

Sagittal images (A, C and E) revealed lytic changes of the adjacent endplates with disruption of the cortical bone and vertebral bodies at the level of T4-T6 (*) and loss of normal disc structure at the level of T5-T6 (arrow). In addition, an elongated extradural T1W hypointense and contrast enhancing lesion consistent with spinal epidural empyema (+) is noted in the ventral aspect of the vertebral canal, extending from T4 to T6 causing moderate compression of the spinal cord. A severe post-contrast enhancing lesion at the level of the paravertebral soft tissues is also shown (^).

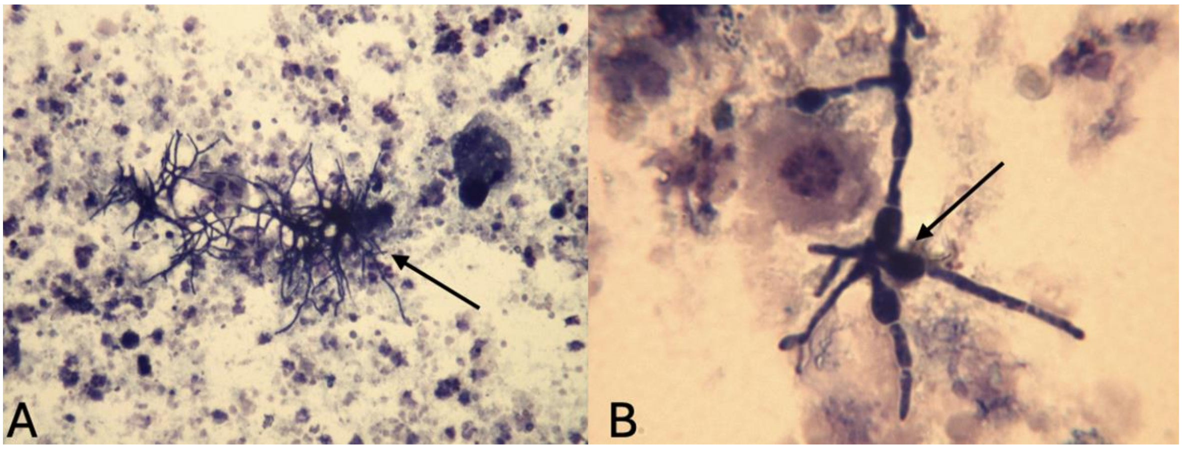

FIGURE 2

Photomicrographs of stained (Wright-Giemsa) cytocentrifugated urine samples at 400x magnification (A) and 1000x magnification (B). Both images show fungal hyphae (arrows).
