# Supplementary material for: Clinical Signs, Advanced Diagnostic Imaging Findings, Treatment, and Outcome of Mycotic Discospondylitis in 11 Dogs
Source: J Vet Intern Med. 2025 Apr 24;39(3):e70097. doi: 10.1111/jvim.70097 (PMC12018769; doi:10.1111/jvim.70097)
Supplement: Supplementary file 2 — Tables S1–S3. [file JVIM-39-e70097-s001.pdf]

**Dog**	**Signalment**	**Onset and progression of neurological signs**	**Neurological status**	**Neuroanatomical localization**
**1**	4.9 yo MN German sheperd	Chronic progressive	Grade 2	T3-L3
**2**	10.9 yo FE Mixed breed	Acute progressive	Grade 4	T3-L3
**3**	6.1yo MN Siberian husky	Chronic intermittent	Grade 3	Multifocal (T3-L3+L4-S3)
**4**	9.3 yo ME German sheperd	Chronic intermittent	Grade 4	T3-L3
**5**	4.0 yo FE German sheperd	Chronic progressive	Grade 3	T3-L3
**6**	3.4 yo ME German sheperd	Chronic progressive	Grade 3	T3-L3
**7**	5.2 yo FN German sheperd	Chronic progressive	Grade 2	T3-L3
**8**	4.7 yo FE Belgian sheperd	Chronic progressive	Grade 1	T3-L3
**9**	6.3 yo FN German sheperd	Subacute progressive	Grade 2	T3-L3
**10**	3.2 yo FE German sheperd	Chronic progressive	Grade 1	T3-L3
**11**	10.0 yo ME Bull terrier	Chronic progressive	Grade 5	T3-L3

TABLE 1

Clinical data including signalment, course of the disease, neurological signs, neurological grading, neuroanatomical localization. ME, male entire; MN, male neutered; FE, female entire; FN, female neutered.

Dog	**MRI**	**CT**	**Radiographs**
**1**	T4-T5,T5-T6,T6-T7 and T4-T7 spinal empyema	NP	T5-T6
**2**	L1-L2, L2-L3	NP	WNL
**3**	L2-L3, L3-L4	NP	L2-L3
**4**	L3-L4	L3-L4	L3-L4
**5**	T8-T9	NP	WNL
**6**	T2-T3,T5-T6,T11-T12,T13-L1,L3-L4,L6-7	T2-T3,T5-T6,T11-T12,T13-L1,L3-4,L6-7	NP
**7**	L1-L2 and L1-L2 spinal empyema	NP	L1-L2
**8**	T3-T4,T6-T7,T8-T9,T10-T11	NP	T3-T4,T6-T7,T8-T9,T10-T11
**9**	T5-T6,T8-T9,T13-L1,L6-L7,L7-S1 and L7-S1 spinal empyema	NP	NP
**10**	Involvement of the entire vertebral column T3-S1, except intervertebral discs L7-S1 and T11-13 and T4-T8 spinal empyema	NP	NP
**11**	T12-T12 and T12-T13 spinal empyema	NP	NP

TABLE 2

Description of the localization of the lesions according to the different diagnostic imaging techniques.

NP, not performed; WNL, within normal limit.

Dog	WBC (x 10^3/µl)	CRP (mg/dL)	Sample for mycological colture	Etiological agent	Main therapy	Additional therapies	Outcome	Survival time (days)
**1**	Unremarkable	Unremarkable	Urine	Aspergillus terreus	/	/	Euthanasia due to concurrent acute kidney injury	21
**2**	Unremarkable	Unremarkable	Urine	Aspergillus fumigatus	Itraconazol	Gabapentin, Tramadol and Antibiotic	Died to unrelated cause (hit by car)	90
**3**	WBC 28.84 (5.05-16.76) neutrophils 21.48 (2.95-11.67)	12.0 (0-0.8)	Material from surgical curettage	Candida albicans	Surgical curettage and Itraconazol	Gabapentin, Tramadol and Antibiotic	Euthanasia due to worsening of ambulatory status	360
**4**	Unremarkable	Unremarkable	Material from surgical curettage	Aspergillus fumigatus	Surgical curettage and Itraconazol	NSAID, Gabapentin, Tramadol and Antibiotic	Euthanasia due to worsening of ambulatory status	5
**5**	WBC 21.12 (5.05-16.76) neutrophils 17.70 (2.95-11.67)	9.7 (0-0.8)	CSF	Unidentified	Itraconazol	AEDs and Antibiotic	Euthanasia due to refractory status epilepticus	10
**6**	WBC 26.7 (5.00-11.00) neutrophils 22.89 (2.95-11.67) monocytes 2.6 (0.16-1.12)	10.4 (0-1.0)	Urine	Aspergillus spp.	Itraconazol	NSAID, Gabapentin, Tramadol and Antibiotic	Alive after 1130 days. Follow-up MRI after 510 days revealed no contrast enhancement of the intervertebral discs	/
**7**	Unremarkable	Unremarkable	CT-guided needle aspiration	Aspergillus spp.	NA	NA	Died due to severe pneumonia	30
**8**	WBC 29.9 (5.00-11.00) neutrophils 23.40 (2.95-11.67) monocytes 2.02 (0.16-1.12)	7.3 (0-1.0)	CT-guided needle aspiration	Saccaromyces spp.	Itraconazol	Gabapentin and Tramadol	Euthanasia due to worsening of ambulatory status. Follow-up MRI after 270 days without contrast enhancement of the intervertebral discs. Second follow-up MRI after 360 with recurrence of discospondylitis	365
**9**	Unremarkable	Unremarkable	Surgical curettage	Penicillum spp.	Surgical curettage and Amphotericin B and Flucytosine	NSAID, Gabapentin and Tramadol	Euthanasia due to worsening of ambulatory status	33
**10**	WBC 17.9 (5.05-16.76) neutrophils 13.10 (2.95-11.64)	8.1 (0-1.0)	Urine	Aspergillus spp.	Fluconazol	NSAID, Gabapentin, Tramadol and Antibiotic	Died of unknow cause	31
**11**	WBC 31.3 (5.05-16.76) neutrophils 27.34 (2.95-11.67)	12.9 (0-0.8)	Urine	Aspergillus spp.	Itraconazol	Gabapentin, Tramadol and Antibiotic	Died due to systemic mycosis	10

TABLE 3

Blood test changes, sample for mycological colture, etiological agent, antifungal and additional therapies, outcome and survival time in our study population. AEDs, antiepileptic drugs; CRP, C-Reactive Protein; CSF, cerebrospinal fluid; CT, computed tomography; NA, not available; NSAIDs, nonsteroidal anti-inflammatory drugs; WBC, White Blood Cells.
